# Use of Deep Learning to Analyze Social Media Discussions About the Human Papillomavirus Vaccine

**DOI:** 10.1001/jamanetworkopen.2020.22025

**Published:** 2020-11-13

**Authors:** Jingcheng Du, Chongliang Luo, Ross Shegog, Jiang Bian, Rachel M. Cunningham, Julie A. Boom, Gregory A. Poland, Yong Chen, Cui Tao

**Affiliations:** 1 School of Biomedical Informatics, The University of Texas Health Science Center at Houston; 2 Perelman School of Medicine, The University of Pennsylvania, Philadelphia; 3 School of Public Health, The University of Texas Health Science Center at Houston; 4 Health Outcomes and Biomedical Informatics, College of Medicine, University of Florida, Gainesville; 5 Texas Children’s Hospital, Houston; 6 Baylor College of Medicine, Houston, Texas; 7 Mayo Clinic Vaccine Research Group, Mayo Clinic, Rochester, Minnesota

## Abstract

**Importance:**

Human papillomavirus (HPV) vaccine hesitancy or refusal is common among parents of adolescents. An understanding of public perceptions from the perspective of behavior change theories can facilitate effective and targeted vaccine promotion strategies.

**Objective:**

To develop and validate deep learning models for understanding public perceptions of HPV vaccines from the perspective of behavior change theories using data from social media.

**Design, Setting, and Participants:**

This retrospective cohort study, conducted from April to August 2019, included longitudinal and geographic analyses of public perceptions regarding HPV vaccines, using sampled HPV vaccine–related Twitter discussions collected from January 2014 to October 2018.

**Main Outcomes and Measures:**

The prevalence of social media discussions related to the construct of health belief model (HBM) and theory of planned behavior (TPB), categorized by deep learning algorithms. Locally estimated scatterplot smoothing (LOESS) revealed trends of constructs. Social media users’ US state–level home location information was extracted from their profiles, and geographic analyses were performed to identify the clustering of public perceptions of the HPV vaccine.

**Results:**

A total of 1 431 463 English-language posts from 486 116 unique usernames were collected. Deep learning algorithms achieved F-1 scores ranging from 0.6805 (95% CI, 0.6516-0.7094) to 0.9421 (95% CI, 0.9380-0.9462) in mapping discussions to the constructs of behavior change theories. LOESS revealed trends in constructs; for example, prevalence of perceived barriers, a construct of HBM, deceased from its apex in July 2015 (56.2%) to its lowest prevalence in October 2018 (28.4%; difference, 27.8%; *P* < .001); Positive attitudes toward the HPV vaccine, a construct of TPB, increased from early 2017 (30.7%) to 41.9% at the end of the study (difference, 11.2%; *P* < .001), while negative attitudes decreased from 42.3% to 31.3% (difference, 11.0%; *P* < .001) during the same period. Interstate variations in public perceptions of the HPV vaccine were also identified; for example, the states of Ohio and Maine showed a relatively high prevalence of perceived barriers (11 531 of 17 106 [67.4%] and 1157 of 1684 [68.7%]) and negative attitudes (9655 of 17 197 [56.1%] and 1080 of 1793 [60.2%]).

**Conclusions and Relevance:**

This cohort study provided a good understanding of public perceptions on social media and evolving trends in terms of multiple dimensions. The interstate variations of public perceptions could be associated with the rise of local antivaccine sentiment. The methods described in this study represent an early contribution to using existing empirically and theoretically based frameworks that describe human decision-making in conjunction with more intelligent deep learning algorithms. Furthermore, these data demonstrate the ability to collect large-scale HPV vaccine perception and intention data that can inform public health communication and education programs designed to improve immunization rates at the community, state, or even national level.

## Key Points

**Question:**

Can public perceptions of the human papillomavirus (HPV) vaccine be accessed from the perspective of behavior change theories by mining social media data with machine learning algorithms?

**Findings:**

This cohort study included 1 431 463 English-language posts about the HPV vaccine from 486 116 unique usernames from a social media platform. An increase in HPV vaccine–related discussions was found, and the results suggest temporal and geographic variations in public perceptions of the HPV vaccine.

**Meaning:**

The findings of this study suggest that social media and machine learning algorithms can serve as a complementary approach to inform public health surveillance and understanding and help to design targeted educational and communication programs that increase HPV vaccine acceptance.

## Teaser

This cohort study develops and validates deep learning models to understand public perceptions of human papillomavirus (HPV) vaccines from the perspective of behavior change theories using data from social media.

## Introduction

Human papillomavirus (HPV) is the most common sexually transmitted disease in the United States.^1^ HPV infections cause approximately 33 700 cases of cancer every year in the United States, including cervical, vaginal, vulvar, penile, and anal cancers.^2,3^ The HPV vaccine has been available since 2006 to protect against HPV-associated cancers and is recommended for adolescents starting at age 9 years through age 26 years if not vaccinated, and, for some people, up to age 45 years.^4^ Unfortunately, compared with other adolescent vaccines (eg, tetanus, diphtheria, pertussis [Tdap] and meningococcal B [MenB]), HPV vaccine rates remain low, with approximately 51% of adolescents not completing the HPV vaccination series.^5^ The most common reasons for parental declination of HPV vaccine include safety concerns, perceived lack of necessity, and lack of knowledge about the vaccine and HPV.^6^ For this reason, knowledge about the prevalence of these concerns can inform tailored strategies to mitigate them and improve immunization rates.

Behavior change theories provide a conceptual framework to understand the determinants of and methods for influencing specific health behaviors.^7^ The health belief model (HBM) and the theory of planned behavior (TPB) are among the most popular behavior change theories that have been widely adopted to explain health behaviors. The HBM assumes that motivation to adopt preventive health behaviors, such as screening and vaccination, is primarily due to the following constructs: perceived susceptibility, perceived severity, perceived benefits, perceived barriers, cues to action, and self-efficacy.^8^ The TPB assumes that constructs, including attitudes, subjective norms, and perceived behavioral control, drive people’s intention to perform a healthy behavior.^9^ Associations have been established between the theoretical constructs of HBM^10,11,12,13^ and TPB^14,15,16^ and HPV vaccination intention and uptake.

Improving understanding of the public perceptions of HPV and the HPV vaccine is essential to developing tailored educational efforts and increasing HPV vaccination rates. Furthermore, understanding these perceptions at the community, state, and national levels over time can provide detailed data useful in designing targeted approaches to improving immunization education programs and public health campaigns. Social media platforms offer a unique opportunity to examine the unfiltered opinions, comments, and discussions of large populations, while mitigating the limitations of traditional surveys, which include resource costs and the difficulties of tracking changes in real-time.^17,18,19^ Our objective was to use machine learning (ML) methods to examine HPV vaccine discussions on Twitter, which has been recognized as 1 of the major sources for accessing public opinions on various topics, from politics^20^ to public health.^21^ Compared with other social media platforms, Twitter has fewer privacy restrictions (ie, easy access to large-scale public discussions) and has younger users than the general population,^22^ which makes it an important resource to study adolescent vaccine-related discussions.

Semiautomatic methods to understand social media vaccine discussions included manual coding and hashtag or keywords analysis,^23,24,25,26^ but these are limited by lack of scalability and inaccuracies, respectively. Given the unique characteristics of the tweet as a social media post (eg, short text, occurrences of cyber slang), obtaining an accurate understanding of these discussions is challenging.^27^ ML methods emerged to address these limitations and to improve the precision of understanding the public perception of vaccines,^28,29^ particularly the HPV vaccine.^30,31,32,33,34,35^ As a subset of ML algorithms, deep learning (DL) algorithms have been applied in analyzing social media natural language processing (NLP) tasks,^36,37,38^ and its superiority has been found in comparison with traditional ML efforts.^39,40^ DL is also advantageous because it can save significant feature engineering efforts in NLP (the process of extracting numeric features from the text that represents the meaning of the contents and is crucial to the effectiveness of these learning algorithms), which is typically required by ML algorithms. A glossary of ML-relevant concepts in this study is provided in the eTable in the Supplement.

## Methods

### Ethics Approval and Consent to Participate

This study received an institutional review board exemption from the Committee for the Protection of Human Subjects at The University of Texas Health Science Center at Houston. A waiver of informed consent was granted due to the retrospective design of the study. This study follows the Strengthening the Reporting of Observational Studies in Epidemiology (STROBE) reporting guideline.

### Study Overview

**Figure 1.**
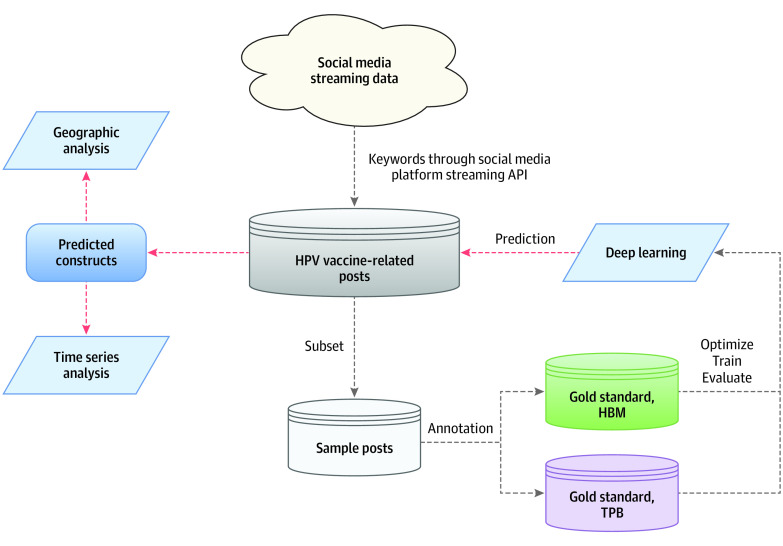
Overview of Study Design API indicates application programming interface; HBM, health belief model; HPV, human papillomavirus; TPB, theory of planned behaviors.

An overview of the study design can be seen in Figure 1. We first collected HPV vaccine–related sampled discussions, using keywords, and then manually categorized (ie, annotated, in the language of ML) a subset of the posts with regard to the theoretical constructs of HBM and TPB. The initial human-categorized posts were the gold-standard corpus^41^ (ie, posts with human-assigned labels) that were used to train and evaluate the ML and DL algorithms. The models that performed most successfully (ie, had the highest F-1 scores) were selected and applied to the remaining unlabeled posts. The analyses, including time-series analysis and geographic analysis, were then performed on the DL-categorized behavioral constructs to identify variations of public perceptions toward the HPV vaccine.

### Data Collection and Initial Human Categorization

We used a set of keywords to collect HPV vaccine–related posts by using Twitter streaming application programming interface (approximately 1% of the entire stream volume) from January 1, 2014, to October 26, 2018. Keywords included *hpv*, *human papillomavirus*, *gardasil*, and *cervarix*. Only English-language posts were included in the study. In regard to HBM, we focused on the 4 primary constructs, including perceived susceptibility, perceived severity, perceived benefits, and perceived barriers; for the TPB model, we focused on an amalgamated construct of attitude. Several other constructs also influence HPV vaccination behavior. However, considering the low prevalence of these constructs in our data set, this study focused only on the major constructs noted earlier.

The human categorizations of the social media platform discussions to HBM or TPM were acquired from our previous studies.^40,42^ The included constructs, definitions, and examples of posts to the social media platform are shown in Table 1. Three reviewers were trained and then categorized a subset of 6000 posts based on their relevance to the HBM constructs. Each post was assigned to none (not related to HBM), 1, or multiple HBM constructs. For TPB constructs, 3 reviewers categorized the same 6000 posts based on its attitude toward the HPV vaccine. The reviewer first decided whether the post was related to the attitude toward the HPV vaccine. If it was related, the reviewer further decided whether it was positive, negative, or neutral. This gold-standard corpus was then used to train and evaluate a variety of ML and DL algorithms. The annotated corpus is available online.^43^

**Table 1.** Definitions and Examples of Key Constructs of Behavior Change Theories Found in a Social Media Platform^a^

Behavior change theory	Construct	Definition	Sample raw posts
Health belief model	Perceived susceptibility	The assessment of the risk of getting an HPV infection	HPV is so common almost everyone will be infected with the virus. but it can cause cancer. so why wait? vaccinate! Men equally at risk of hpv infection: boys should also be vaccinated for the human papillomavirus
	Perceived severity	The assessment of whether an HPV infection is a sufficient health concern	Learn about the human papillomavirus, which causes almost all cases of cervical cancer HPV is more badass than HIV. keep yourself
	Perceived benefits	Benefits of the HPV vaccine in protecting against HPV infection, HPV infection-induced cancers, and so forth	Health lifestyle | here's how the hpv vaccine can help cut the risk of cancer in gay men news &gt; Benefits of hpv vaccine can be seen in high school girls, study says
	Perceived barriers	Adverse effects of the HPV vaccine, cost of getting an HPV vaccine, negative news and reports on the HPV vaccine, and so forth	HPV vaccine is associated with serious health risks HPV vax kills a much higher % of young athletic girls
Theory of planned behavior	Positive attitude	Shows positive opinion or prompt HPV vaccine	Save lives by getting children the HPV vaccine The HPV vax prevents cancers later in life
	Neutral attitude	Related to HPV vaccine topic but contains no sentiment or sentiment is unclear	About 1 of 10 NJ boys received all 3 doses of HPV vaccine The myths and facts about HPV vaccines
	Negative attitude	Concerns or doubts about the HPV vaccine	Study reveals ‘unaoidable’ danger of HPV vaccines Just seen a story, girl got her hpv vaccination, starting having seizures so bad she couldn’t stay in school anymore, her junior year!

Abbreviation: HPV, human papillomavirus.

^a^ Some definitions and examples were from our previous studies.^40,42^

### DL-Based Categorization of Discussions

We framed the automated understanding of content from the social media platform to text classification tasks, which aimed to classify the content of posts to predefined categories. We built ML and DL classifiers for constructs of HBM and TPB, respectively. These classifiers were trained using the human-categorized posts described earlier. For the 4 primary HBM constructs, we first categorized the post based on its relevance to any of the HBM constructs and then categorized the relevant posts to the primary HBM constructs, using binary classification (1 classifier for 1 construct). For TPB constructs, we first categorized the post based on its relevance to attitude toward the HPV vaccine and then categorized the relevant posts into 1 of 3 attitudes: positive, negative, or neutral. Each post was categorized to HBM and TPB separately.

To select the best classifiers for our tasks, we performed an evaluation of multiple ML and DL algorithms and configurations. The descriptions of those algorithms and experimental details are described in the eAppendix in the Supplement. We evaluated the algorithms on HBM and TPB categorization (ie, classification). For each categorization task, we divided task-relevant labeled posts into training, validation, and test sets with a proportion of 7:1:2. We trained the models on the training set, performed hyperparameter selection on the validation set, and evaluated the performance of classifiers on the test set. We repeated random sampling of the posts 10 times (with replacements) with the same proportion and calculated metrics for each model at each time.

For all binary classifiers (ie, classifying the post as HBM related, TPB related, or to each HBM construct), we calculated sensitivity, specificity, accuracy, precision, recall, and F-1 score. For the multiclass classifier (ie, to classify the post into 1 of 3 attitudes), we calculated overall accuracy as well as precision, recall, and F-1 score for each attitude (ie, positive, negative, or neutral).

### Statistical Analysis

All statistical tests were 2-tailed, and statistical significance was set at *P* < .05. Time-series and geographic analyses were conducted in R version 3.6.2 (R Project for Statistical Computing).

#### Temporal and Geographic Analyses of Social Media Discussions

We performed time-series analysis on the predicted constructs to extract the evolving trends and geographic analysis to identify the US interstate variations of public perceptions of the HPV vaccine. We selected the best-performing model (ie, attentive recurrent neural network [Att-RNN] with fastText [FT] HPV embedding; eAppendix in the Supplement) for prediction of the unlabeled data in our collection. To reduce the variances of the DL model,^44^ we repeated random sampling and the training of Att-RNN model 10 times; the final prediction of all the unlabeled posts was based on the majority voting of the 10 models.

#### Time-Series Analysis

We defined the prevalence of each theoretical construct by calculating the proportion of the number of posts that were classified to that construct to the total number of posts that were classified to the corresponding theory. We calculated the prevalence of each construct for each week. To extract the trend of the constructs, we applied time-series analyses to the weekly prevalence data. Specifically, we decomposed the prevalence into seasonal, trend, and random noise components using locally estimated scatterplot smoothing (LOESS).^45^ The decomposition was done by the R function stl. Seasonal-trend decomposition via LOESS smoothing is a common time series analysis method in various disciplines.^45^ We tested the increasing or decreasing trend of each construct by 2-sample proportion test.

#### Geographic Analysis

Users could self-report their home location in their profiles. Because it is optional for users to complete their profiles, the home location information is often sparse. Given that the available home location is in the free-text format, we leveraged an open-source lexicon-based script^46^ to map the home location string to a US state. For example, “Miami, FL” was mapped to Florida, “Texas” to Texas. After excluding the posts for which we could not map users’ home location to a US state, we calculated the count and prevalence of theory and construct-related posts for each US state.

## Results

A total of 1 431 463 English-language posts from 486 116 unique usernames were collected as our study cohort. A total of 6000 posts were selected for the initial human-categorization of discussions on the social media platform. The κ interannotator agreement for each HBM construct ranged from 0.727 to 0.834. The overall κ interannotator agreement TPB categorization was 0.851.

### Performance of Classification Algorithms

The comparison of various word-embedding techniques and classification algorithms can be seen in the eAppendix in the Supplement. The DL model Att-RNN with FT HPV word embedding provided the best performance on most tasks and was thus selected for prediction purposes. The performance of Att-RNN with FT HPV word embedding on the gold-standard corpus can be seen in Table 2. The model achieved a mean accuracy of 0.8018 (95% CI, 0.7924-0.8113) and 0.9226 (95% CI, 0.9171-0.9281) for identifying HBM-related and TPB-related posts, respectively. For HBM-related constructs, the model achieved a mean accuracy between 0.8721 (95% CI, 0.8614-0.8828) and 0.9063 (95% CI, 0.8977-0.9149) and a mean F-1 score between 0.6805 (95% CI, 0.6516-0.7094) and 0.8999 (95% CI, 0.8906-0.9091). For identifying TPB-related posts, the model achieved a mean F-1 score of 0.9421 (95% CI, 0.9380-0.9462); for TPB attitude, it achieved a mean F-1 score between 0.6996 (95% CI, 0.6841-0.7141) and 0.8103 (95% CI, 0.8011-0.8196).

**Table 2.** Metrics of Att-RNN With FT HPV Embedding in Mapping Discussions on a Social Media Platform to the Theoretical Constructs

Theory and construct	Mean (95% CI)					
	Sensitivity	Specificity	Accuracy	Precision	Recall	F-1 score
Health belief model						
Related	0.8072 (0.7823-0.8321)	0.7954 (0.7727-0.8181)	0.8018 (0.7924-0.8113)	0.8254 (0.8120-0.8389)	0.8072 (0.7823 - 0.8321)	0.8156 (0.8049-0.8263)
Susceptibility	0.6889 (0.6489-0.7289)	0.9396 (0.9256-0.9536)	0.9015 (0.8906-0.9125)	0.6784 (0.6276-0.7293)	0.6889 (0.6489 - 0.7289)	0.6805 (0.6516-0.7094)
Severity	0.7620 (0.7194-0.8047)	0.9419 (0.9286-0.9552)	0.9063 (0.8977-0.9149)	0.7681 (0.7322-0.8040)	0.7620 (0.7194 - 0.8047)	0.7626 (0.7405-0.7847)
Benefits	0.7305 (0.6801-0.7808)	0.9197 (0.9043-0.9350)	0.8721 (0.8614-0.8828)	0.7564 (0.7281-0.7846)	0.7305 (0.6801 - 0.7808)	0.7407 (0.7154-0.7661)
Barriers	0.8890 (0.8682-0.9098)	0.9219 (0.9017-0.9420)	0.9063 (0.8975-0.9150)	0.9123 (0.8929-0.9317)	0.8890 (0.8682 - 0.9098)	0.8999 (0.8906-0.9091)
Theory of planned behavior						
Related	0.9487 (0.9429-0.9546)	0.8710 (0.8568-0.8851)	0.9226 (0.9171-0.9281)	0.9357 (0.9291-0.9422)	0.9487 (0.9429 - 0.9546)	0.9421 (0.9380-0.9462)
Positive	NA	NA	NA	0.7425 (0.7144-0.7705)	0.7500 (0.7201-0.7798)	0.7447 (0.7307-0.7587)
Negative	NA	NA	NA	0.7987 (0.7842-0.8132)	0.8235 (0.8007-0.8464)	0.8103 (0.8011-0.8196)
Neutral	NA	NA	NA	0.7172 (0.6992-0.7351)	0.6843 (0.6579-0.7106)	0.6996 (0.6841-0.7151)

Abbreviations: Att-RNN, attentive recurrent neural network; FT, fastText; HPV, human papillomavirus; NA, not applicable.

### Temporal Trends for Theoretical Constructs

After applying the models to classify the unlabeled posts, 948 501 and 920 486 posts were classified as HBM related and TPB related, respectively. For HBM-related posts, 125 516 (13.2%), 215 964 (22.8%), 239 835 (25.3%), and 387 049 (40.8%) were classified into susceptibility, severity, benefits, and barriers, respectively. For TPB attitude–related posts, 331 836 (36.1%); 341 281 (37.1%), and 247 369 (26.9%) were classified into positive, negative, and neutral, respectively.

**Figure 2.**
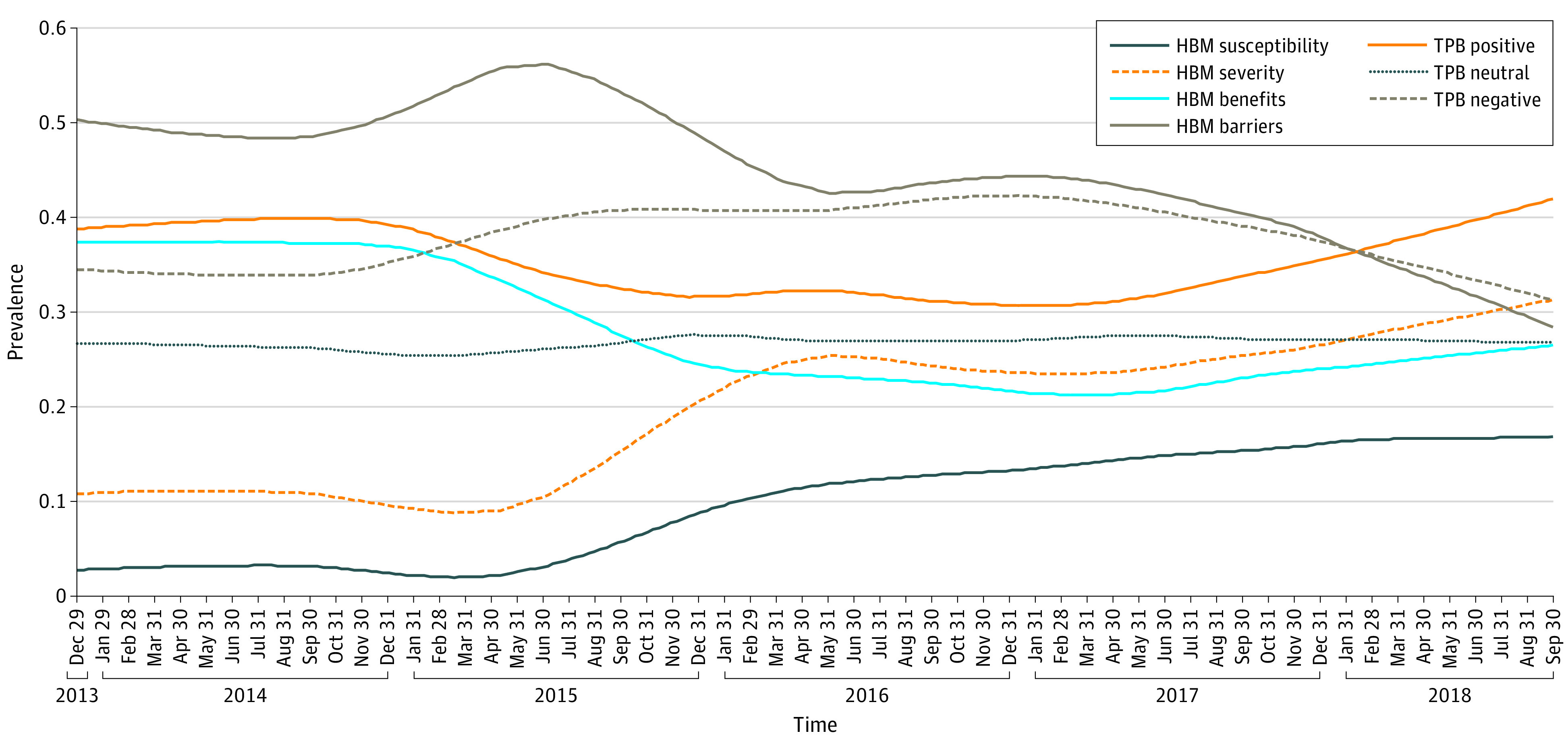
Trend of Theoretical Constructs After Removing Seasonal Effect and Random Noise HBM indicates health belief model; TPB, theory of planned behaviors.

There were dramatic fluctuations in the prevalence of each construct (eFigure 1 and eFigure 2 in the Supplement). In addition, there were increasing trends for the total number of theory-related posts (ie, HBM related and TPB related) during the study period. Time-series analysis further extracted smooth trends for each construct (Figure 2). Among HBM-related constructs, there was a decreasing trend for the prevalence of barriers, from its highest peak in July 2015 (56.2%) to the lowest prevalence in October 2018 (28.4%; difference, 27.8%; *P* < .001). We also found an increasing trend for the prevalence of severity, with the lowest prevalence in March 2015 (8.8%) and the highest prevalence in October 2018 (31.3%; difference, 22.5%; *P* < .001). The prevalence of benefits decreased from early in 2015 to the middle of 2016 and remained relatively stable afterward; susceptibility demonstrated an opposite trend, as the prevalence increased from early 2015, with the lowest prevalence in March 2015 (1.9%) and highest prevalence in September 2018 (16.8%; difference, 14.9%; *P* < .001). Among the attitudes toward the HPV vaccine, neutral attitude stayed stable over the years; since early 2017, positive attitude toward the HPV vaccine demonstrated an increasing trend, from 30.7% to 41.9% (difference, 11.2%; *P* < .001), while negative attitude demonstrated a decreasing trend, from 42.3% to 31.3% (difference, 11.0%; *P* < .001).

### Interstate Variations of HPV Vaccine Perceptions

**Figure 3.**
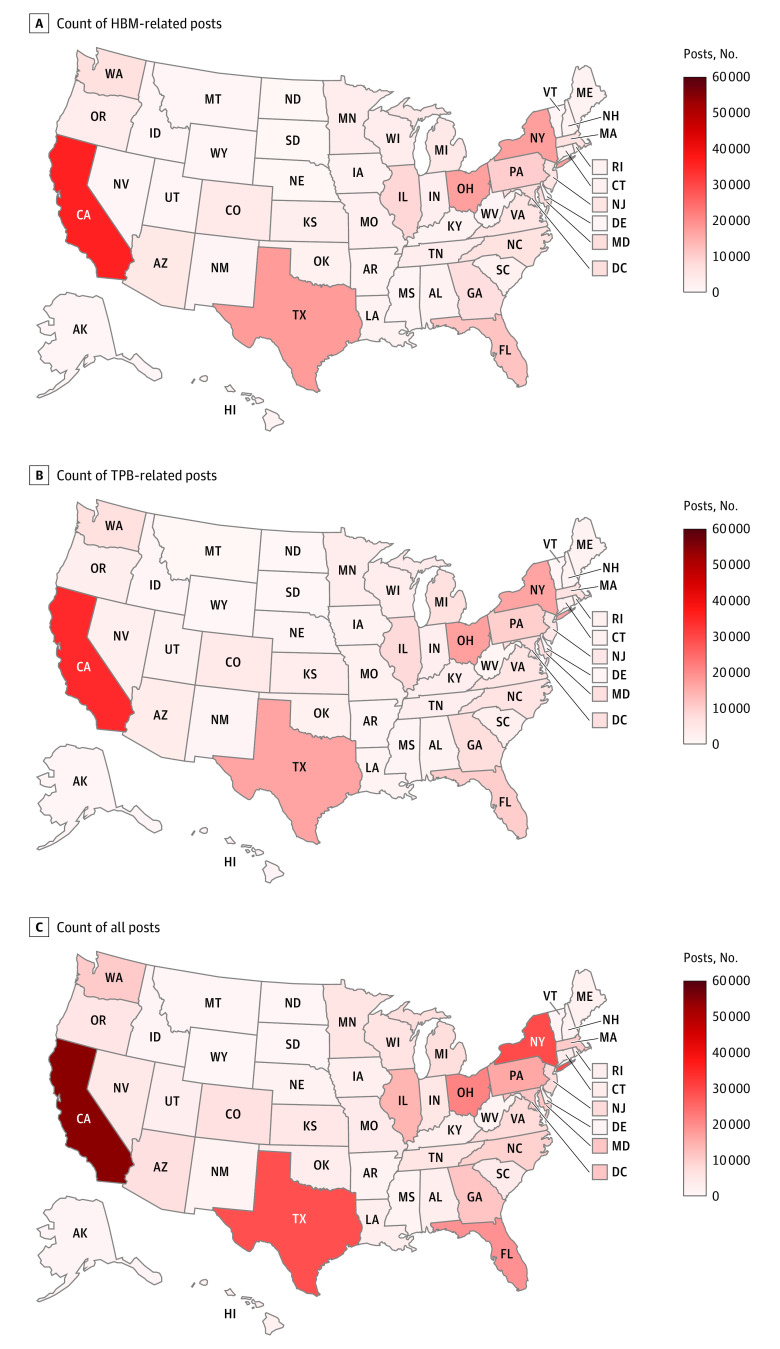
Interstate Variations on the Count of HPV Vaccine–Related Discussions on Social Media HBM indicates health belief model; TPB, theory of planned behaviors.

There were 486 116 unique usernames derived from 1 431 463 contributions to the platform. Among these users, 128 812 profiles (26.5%) (369 181 posts [25.8%] in total) had home locations that could be mapped to US states. The geographical analyses of HPV vaccine perceptions were based on these 369 181 posts. Figure 3 shows the clustering of HPV vaccine discussions. HPV vaccine–related discussions were clustered in US states with large populations. California had the largest proportion of HPV vaccine–related discussions on the site (54 764 of 369 181 [14.8%]). Other large US states, such as Texas, New York, Ohio, and Florida, also show clustered discussions related to the HPV vaccine.

We further examined the interstate variations on the prevalence of theoretical constructs regarding the HPV vaccine (eFigure 3 and eFigure 4 in the Supplement). For HBM constructs, states in the central US, including South Dakota, Nebraska, and Kansas, showed a relatively higher prevalence of discussions related to perceived benefits (144 of 357 [40.3%], 434 of 996 [43.6%], and 1191 of 3033 [39.3%], respectively) and a relatively lower prevalence of perceived barriers (79 [22.1%], 178 [17.9%], and 650 [21.4%], respectively). In particular, Ohio and Maine showed a high prevalence of discussions related to perceived barriers (11 531 of 17 106 [67.4%] and 1157 of 1684 [68.7%], respectively) and low prevalence of discussions related to perceived benefits (2057 [12.0%] and 212 [12.6%], respectively). For TPB attitude, similar to HBM constructs, states in the central United States showed a relatively higher prevalence of discussions related to positive attitudes toward the HPV vaccine and a relatively lower prevalence of discussions related to negative attitudes. In particular, Ohio and Maine demonstrated a high prevalence of negative attitudes (9655 of 17 197 [56.1%] and 1080 of 1793 [60.2%], respectively).

## Discussion

Vaccine hesitancy is listed as among the top 10 global health threats by the World Health Organization (WHO).^47^ Existing studies have found that HPV vaccine refusal or hesitancy may be motivated by theoretical constructs of behavior change theories.^11,48^ In this study, we examined social media trends related to HPV and the HPV vaccine in connection with the HBM and TPB constructs. We found an increase in the number of theory-related posts (ie, HBM related and TPB related) during the years of our study, demonstrating an increased interest in discussing the HPV vaccine on social media. Overall, our findings suggest public perception of the HPV vaccine may be improving. We found that attitudes toward the HPV vaccine became more positive in recent years. This may be attributable to the substantial efforts put forth by the medical and public health community regarding HPV and the HPV vaccine. We also found an increase in users’ perception of HPV severity, which demonstrates that a shift to a focus on cancer prevention in regard to HPV has been effective. After the licensure of the HPV vaccine and the slow acceptance among parents of adolescents, the US Centers for Disease Control and Prevention (CDC) shifted their educational efforts and messaging to focus primarily on HPV vaccination as cancer prevention. They also encouraged health care professionals to issue strong presumptive recommendations and bundle all recommended adolescent vaccines together rather than singling out HPV, regardless of it not being required by many states for school entry. Our study demonstrates that the surveillance of social media discussions regarding vaccines could assist communication in responses to the rise of antivaccine sentiment in a timely manner, inform educational efforts, and gauge national opinion in regard to HPV vaccine. In addition, our approach enables us to understand an individual’s health beliefs and attitudes toward the vaccine, which could facilitate further innovative and customized vaccination promotion strategies.

The analyses of public perception variations in certain states could assist public health professionals mitigate the influence of local antivaccine movements, examine vaccine policy, and inform vaccine-promotion campaigns. The clustering of antivaccine sentiment (eg, high prevalence in perceived barriers and negative attitudes) could relate to the rise of local antivaccine sentiment. For example, in the present study, Ohio was identified as having a higher prevalence of antivaccine sentiment on social media. A review of contributions to the social media platform from Ohio residents found that most discussions from this state regarding the HPV vaccine related to rumors and misinformation about the injuries and risks associated with the HPV vaccine. The clustering of antivaccine sentiment regarding the HPV vaccine in states such as Ohio also could be promoted by the local antivaccine movement^49^ and antivaccine thought influencers^50^ who reside in these states. Health care professionals have increasingly reported social media as a major source of information cited by parents against HPV vaccination.^51^ For health care professionals, trends in social media discussions within specific communities, states, or regions could help to predict future patient sentiment and alert a practice to expect and prepare for potentially increased levels of vaccine hesitancy. This would give health care professionals the opportunity to engage in training and education for vaccines, if needed, and to establish practices within their offices to address vaccine hesitancy and/or refusal.

This study is important in the context of population-level and individual-level vaccination decision-making. Social media surveillance can assist in understanding popular trends in opinion, alerting public health practitioners to the pulse of public sentiment. Significant for public health is the potential to intelligently process social media messaging, categorizing the sender’s motivations (ie, HBM and TPB construct–related perceptions) and interjecting salient, tailored commentary as a counterfactual to misinformation. The methods described in this study may enable persuasive messaging to be injected into social media streams to mitigate vaccine hesitancy in the general public and, more pointedly, among parents of vaccine-eligible children. This intervention strategy offers the potential for future research and may assist in reducing vaccine hesitancy and thus contribute to the mission of pediatric and adolescent practices in achieving HPV vaccination goals. The methods described in this study represent an early contribution to using existing empirically and theoretically based frameworks to develop more intelligent artificial intelligence and DL algorithms that may positively influence HPV vaccine decision-making.

At the current time, public health departments rely on slow, expensive, and time-limited methods, such as paper or electronic surveys or occasional large-scale studies designed for other reasons but that collect vaccine-related data (eg, national behavioral health surveys). Such methods are characterized by long lag times between survey and vaccine decision-making, rely on respondent recall, and provide only gross summary metrics without targeted and regionally actionable information. This study demonstrated the feasibility of methods that benefit vaccine-promotion programs. It provided a method to automatically understand population-level and individual-level health beliefs and attitudes toward the HPV vaccine. This can then inform rational and directed programmatic efforts to improve actual immunization coverage rates by allowing for real-time monitoring of beliefs and intentions and adjustment of educational and public health campaigns and messaging as warranted. Such data-enabled real-time information is invaluable to the design of such efforts and can assist in realizing the benefits of increased population vaccine coverage levels.

### Limitations

There are a few limitations on public health surveillance using social media.^52^ Particular to this is population bias, ie, social media users may not be representative of the general population. Thus, findings based on social media data should be interpreted with caution. However, as the population of users on the social media platform we studied tends to be younger than the general population,^53^ which is the target population for HPV vaccine promotion, we believe the public opinions on this platform can be very valuable and complementary to traditional survey-based findings. Another limitation of this study is that the treatment of predicted labels as true labels for the time-series analysis could lead to information bias due to misclassification rates.^54,55^ Given that the models achieved high accuracy on most tasks, we believe that the general trends are reliable. A further limitation is a gold-standard corpus limited to 6000 posts. This may not fully represent the unlabeled collection (approximately 1.5 million posts), and the shift in the data distribution between labeled and unlabeled data might bring additional bias to the prediction. To mitigate this, we recommend that future studies add more representative posts to the gold-standard corpus.

## Conclusions

This study evaluated DL algorithms for mapping HPV vaccine–related social media discussions to the constructs of behavior change theories. DL algorithms outperformed ML algorithms on our tasks. In particular, the study provided data demonstrating several important parameters useful to designing strategies that could improve immunization coverage rates. First, time-series analysis on the predicted constructs revealed the evolving trends of public perception in regard to the HPV vaccine. Second, geographical analyses identified state-level clustering of public perceptions in regard to the HPV vaccine. This is important in terms of understanding the epidemiology of vaccine misinformation and disinformation and in targeting geographic areas that need data-informed educational and other programmatic efforts to counter such concerns. Third, this study’s innovation in categorizing messages informed by theory-based constructs to differentiate and fine tune attitudes provided a sound theoretical basis for future public health messaging and for rapidly measuring and assessing the effects of such messaging and programs.

## Notes

Supplement. **eFigure 1. **Prevalence of Constructs From HBM **eFigure 2. **Prevalence of Attitudes From TPB **eFigure 3. **Interstate Variations on the Prevalence of HBM Constructs on Social Media **eFigure 4. **Interstate Variations on the Prevalence of TPB Attitude on Social Media **eTable. **Glossary on Artificial Intelligence and ML-Relevant Concepts Used in This Study **eAppendix. **Supplemental Methods
