# Supplementary material for: Use of Deep Learning to Analyze Social Media Discussions About the Human Papillomavirus Vaccine
Source: JAMA Netw Open. 2020 Nov 13;3(11):e2022025. doi: 10.1001/jamanetworkopen.2020.22025 (PMC7666426; doi:10.1001/jamanetworkopen.2020.22025)
Supplement: Supplement. — eFigure 1. Prevalence of Constructs From HBM eFigure 2. Prevalence of Attitudes From TPB eFigure 3. Interstate Variations on the Prevalence of HBM Constructs on Social Media eFigure 4. Interstate Variations on the Prevalence of TPB Attitude on Social Media eTable. Glossary on Artificial Intelligence and ML-Relevant Concepts Used in This Study eAppendix. Supplemental Methods [file jamanetwopen-e2022025-s001.pdf]

Supplemental Online Content

Du J, Luo C, Shegog R, et al. Use of deep learning to analyze social media discussions about the human papillomavirus vaccine. *JAMA Netw Open*. 2020;3(11):e2022025. doi:10.1001/jamanetworkopen.2020.22025

eFigure 1. Prevalence of Constructs From HBM

eFigure 2. Prevalence of Attitudes From TPB

eFigure 3. Interstate Variations on the Prevalence of HBM Constructs on Social Media

eFigure 4. Interstate Variations on the Prevalence of TPB Attitude on Social Media

eTable. Glossary on Artificial Intelligence and ML-Relevant Concepts Used in This Study

eAppendix. Supplemental Methods

This supplemental material has been provided by the authors to give readers additional information about their work.

© 2020 Du J et al. *JAMA Network Open.*

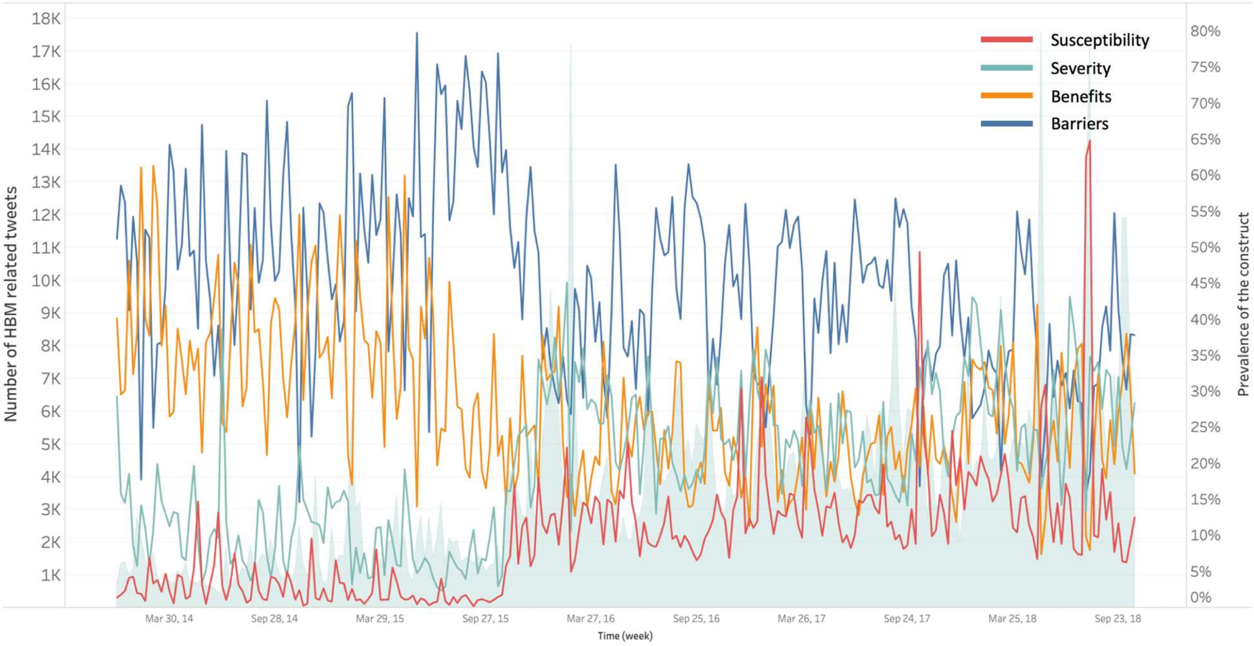

eFigure 1. Prevalence of Constructs From HBM.

The green shadowed area represents the total number of HBM-related tweets each week, and the colored lines represent the prevalence of each construct.

© 2020 Du J et al. *JAMA Network Open.*

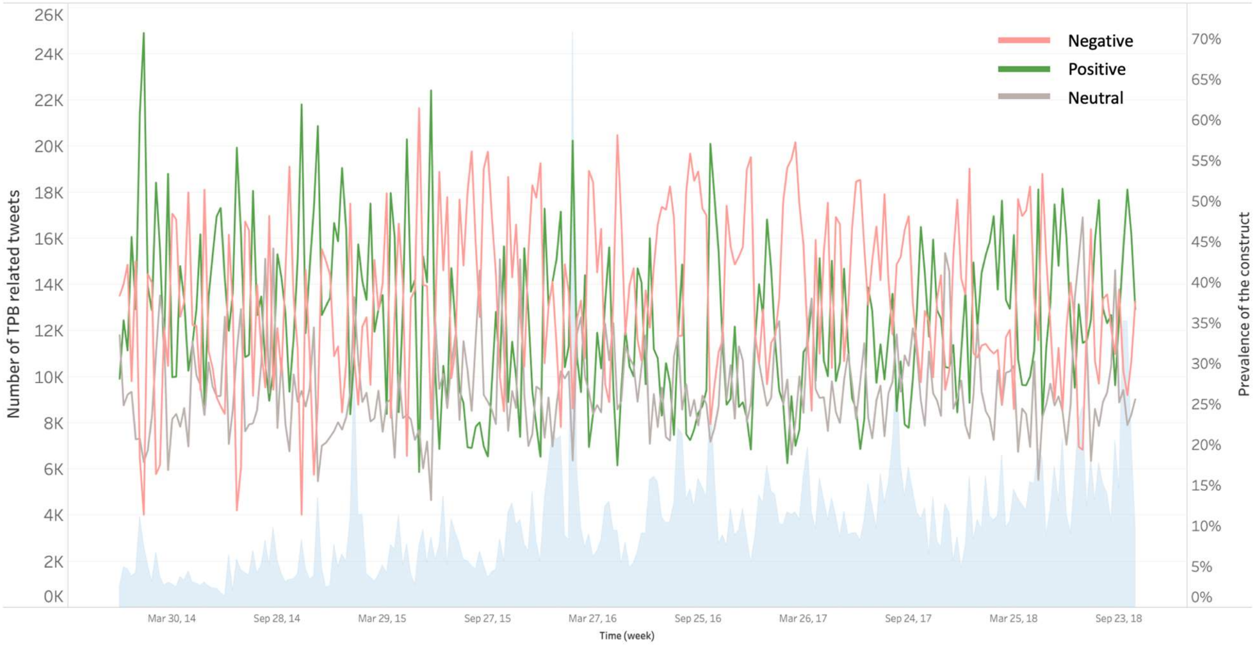

eFigure 2. Prevalence of Attitudes From TPB

The blue shadowed area represents the total number of TPB-related tweets each week, and the colored lines represent the prevalence of each attitude.

© 2020 Du J et al. *JAMA Network Open.*

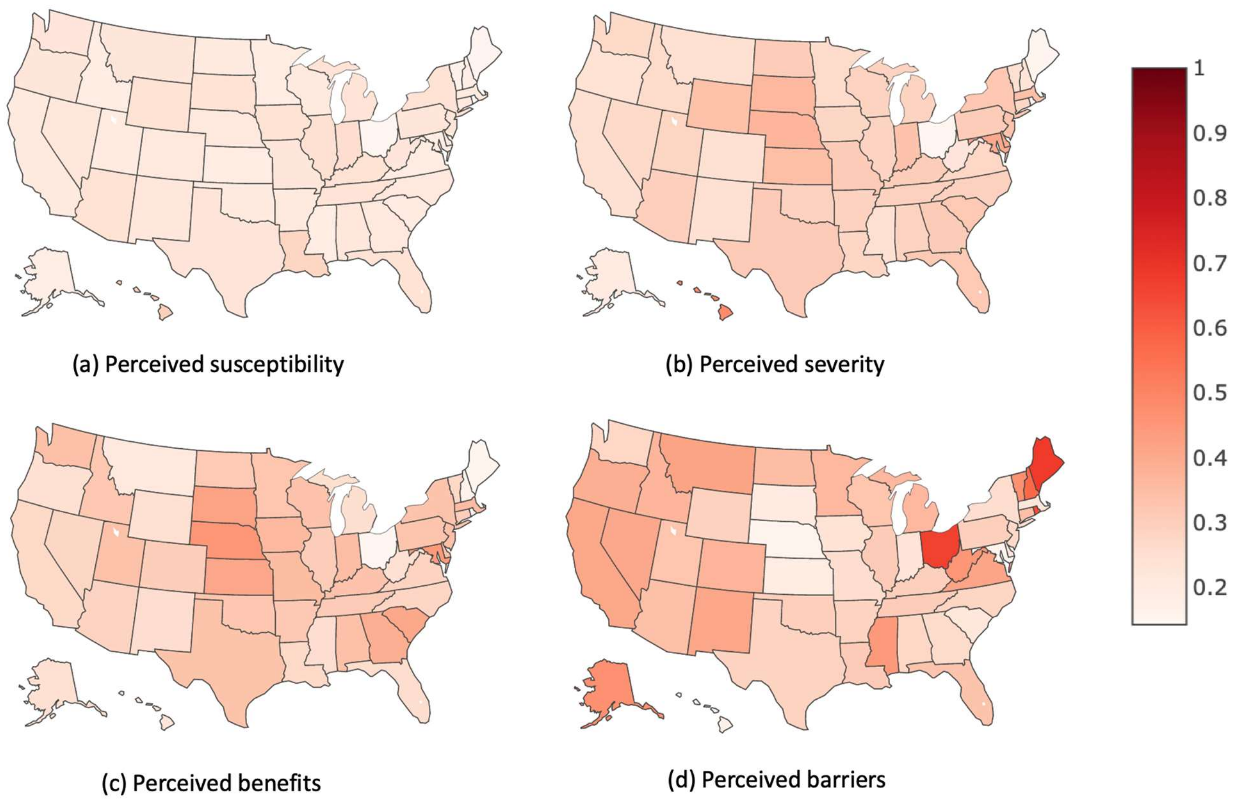

eFigure 3. Interstate Variations on the Prevalence of HBM Constructs on Social Media

© 2020 Du J et al. *JAMA Network Open.*

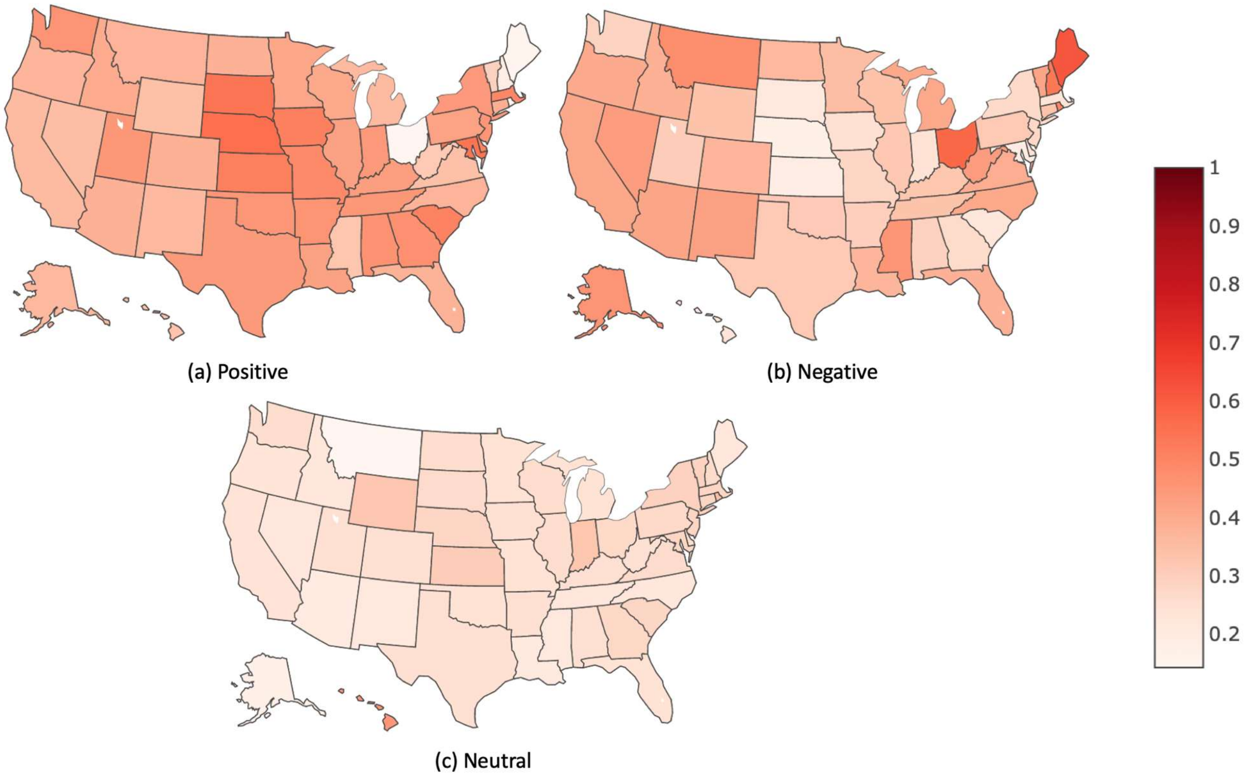

eFigure 4. Interstate Variations on the Prevalence of TPB Attitude on Social Media

© 2020 Du J et al. *JAMA Network Open.*

eTable. Glossary on Artificial Intelligence and ML-Relevant Concepts Used in This Study

**Term**	**Explanation**
Artificial Intelligence	AI is a wide branch of computer science that builds intelligent machines capable of performing tasks that typically require human intelligence.
Natural Language Processing (NLP)	NLP is a field of AI that understands natural language through computers.
Gold Standard Corpus	A robust dataset used for training and evaluation of computational algorithms that have been manually curated by domain experts.
Machine Learning (ML)	ML is an application of AI that provides computers the ability to learn and improve through experience without being explicitly programmed.
Feature Engineering	Feature engineering is the process of extracting numeric features from the original data that can be directly processed by ML algorithms.
Deep Learning (DL)	DL is a subfield of ML that is based on artificial neural network algorithms.
Recurrent Neural Network (RNN)	RNN is a DL model that contains loops within the network which allows information to be stored.
Long Short-Term Memory (LSTM)	LSTM is a variation of RNN that helps to alleviate the vanishing gradient problem by using the "gate" mechanism to retain "memories" of prior time steps.
Word Embedding	A word embedding is a learned word representation that maps the words to high-dimensional vectors, where similar words have similar encodings.
Softmax Layer	Softmax layer is typically the final output layer in a deep learning model that performs multi-class classification. Softmax layer uses softmax function to generate a probability distribution for all classes.
Attention Layer	An attention layer is an effective component of a deep neural network that is selectively concentrating on a few relevant things in a sequence output.

© 2020 Du J et al. *JAMA Network Open.*

eAppendix. Supplemental Methods

Given the unique characteristics of the Twitter text (e.g., short text, occurrences of cyber slang), an accurate understanding of Twitter discussions is considered challenging.[1] Conventional machine-learning-based approaches with feature engineering are very popular for Twitter text classification tasks. Feature engineering in NLP is the process of extracting numeric features from the text that represents the meaning of the contents. As most of the machine learning algorithms are not able to take narrative text directly as the input, feature engineering is crucial to the effectiveness of these learning algorithms. Conventional machine learning algorithms heavily rely on task-specific feature engineering and faces challenges in capturing context information in a tweet text.

Deep learning algorithms have achieved state-of-the-art performance on many text classification tasks.[2–5] Deep learning leverages pre-trained word embedding (i.e. mapping the words to high-dimensional vectors)[6] to capture the necessary semantics of Twitter words, saving significant effort in feature engineering while improving the performance. Pre-trained word embedding learns dense representations of the words from a large collection of textual documents in which similar words have similar encodings[7] A sequence-based deep-learning model, such as the bi-directional recurrent neural network (RNN)[8], can further capture forward and backward information of the corresponding tweet text.

We leveraged a deep-learning framework, *Att-RNN*, which is based on a recurrent neural network[9] as the text classifiers. *Att-RNN* consists of several layers, including a word-embedding layer to map a tweet token (i.e., word) to a high-dimensional vector, a bidirectional long short-term memory layer to capture both forward and backward information, an attention layer to augment the sequence model, and a Softmax output layer for classification. To optimize the

© 2020 Du J et al. *JAMA Network Open.*

performance of the *Att-RNN*, we performed an evaluation of multiple word-embedding approaches and conducted a comparison of multiple competitive machine-learning and deep-learning algorithms.

We've performed extensive experiments to improve the performance of classification algorithms in mapping Twitter discussions to the constructs of behavior change theories, including the evaluation of various Twitter word embeddings, and the evaluation of both traditional machine learning and deep learning algorithms.

Evaluation on the use of Twitter word embeddings

Many machine-learning and almost all deep-learning algorithms are incapable of processing strings and text in their raw form. Pre-trained word embedding, which provides distributed representations of words in a vector space, can help ML algorithms achieve better performance in natural language processing tasks.[10] Various word embedding models have been proposed in recent years, including *word2vec*,[10] *GloVe*,[11] and *fastText*.[12]

word2vec is one of the most popular techniques to learn word embedding There are two main training algorithms for *word2vec*: continuous bag of words (CBOW) and skip-gram. CBOW uses the context of the word to predict a target word while skip-gram uses a word to predict a target context. For the present study, skip-gram was chosen as the algorithm as it works better for infrequent words.[13]

GloVe stands for global vectors for word representation. *GloVe* was proposed by Pennington et al as a count-based method to learn word vectors. Different from *word2vec,* which leverages the predictive model (i.e. neural network) to learn word vectors, *GloVe* learns word vectors from aggregated global word-word co-occurrence.

© 2020 Du J et al. *JAMA Network Open.*

fastText is a more recent method of word embedding, which is based on a skip-gram model. However, contrary to *word2vec*, where the morphology of words is ignored, each word in *fastText* is represented as a bag of character n-grams. A word vector representation is associated with each character n-grams.

Twitter word embedding was trained by applying the above three models to the un-labeled HPV-related Twitter corpus (~1.4 million tweets), which were termed W2V HPV, GloVe HPV, and FT HPV. For all three models, the window size was set at 5, the maximum iteration at 20, and dimension size at 200. The use of these Twitter word embeddings was evaluated on a recurrent neural network with attention mechanism (*Att-RNN*).[9] For comparison purposes, the use of pre-trained 200-dimension GloVe Twitter embedding (trained 2 billion tweets from the general domain, which we term GloVe General) and the use of random 200-dimension embedding were also evaluated.

The performance of the different word-embedding techniques can be seen in eMethods Table 1 – 3. The use of pre-trained Twitter word embedding boosted overall performance compared with the use of randomly initialized embedding. The present study trained domain-specific embedding on a relatively small corpus (i.e., HPV vaccine-related Twitter corpus, which provided comparable and better performance with the general Twitter embedding (GloVe Twitter), trained on billions of tweets on the tasks. In particular, the use of FT HPV embedding led to the best performance in the majority of the tasks. Although domain-specific embedding might not be able to capture all of the necessary semantics, it can be more representative of specific domain-related tasks.[14]

Evaluation of classification algorithms

© 2020 Du J et al. *JAMA Network Open.*

In addition to *Att-RNN*, to demonstrate the superiority of the proposed model, we further evaluated several competitive traditional machine learning algorithms and two deep learning-based algorithms (i.e. *Att-ELMo* and *BERT*) as comparisons.

Several classic machine learning algorithms (e.g., support vector machines, logistic regression, random forest) were tested and extremely randomized trees were chosen (ERT)[15] as the baseline algorithm due to its better performance on most of the tasks. Two types of features were evaluated: (1) mean-embedding - all of the tokens were mapped to high-dimensional vectors using pre-trained word embedding (FT HPV embedding was used in this study) and took the averaged word vectors for all words in each tweet as the feature (which was termed *mean-emb*) and (2) term frequency-inverse document frequency (TF-IDF) - TF-IDF is a numerical statistic that is intended to reflect how important a word is to a document in a corpus.[16]

Att-ELMo is an attentive sequence model based on the Embeddings from Language Models (ELMo).[17] Traditional word embedding methods assign a static high dimensional vector to a word, regardless of its context. However, a word could have multiple context-dependent meanings. ELMo is a deep contextualized word embedding method that can look at the entire context before assigning each word its embedding vector. *Att-ELMo* first adopts the pre-trained ELMo (which was loaded from https://tfhub.dev/google/elmo/2) to map each word in the tweet to high-dimensional vectors. Then, similar to *Att-RNN*, word vectors are then fed to a bidirectional RNN, followed by the attention mechanism. A Softmax layer serves as the output layer for classification.

BERT stands for Bidirectional Encoder Representations from Transformers. *BERT* is a new language representation model based on *Transformer* architecture.[18] *Transformer* relies entirely on self-attention to compute representations of its input and output without using

sequence-aligned RNNs (e.g. LSTM).[19] Contrary to recurrent models, *Transformer* allows for significantly more parallelization. *BERT* achieved state-of-the-art performance in many natural language processing tasks.[18] A pre-trained *BERT* model can be fined tuned with just one additional layer to other tasks. The pre-trained *BERT* model (BERT-Large, Uncased) was loaded and fine-tuned in the present study's Twitter text classification tasks.

The comparison of different classification algorithms can be seen in eMethods Table 4 – 6. Machine-learning algorithms with the TFIDF feature provide relatively strong baseline performance on most of the tasks. TFIDF feature achieved higher accuracy and F-1 score than did the same learning algorithm with the mean-emb feature. In general, deep-learning algorithms (e.g., *Att-RNN*, *Att-ELMo, BERT*) demonstrated superiority over machine-learning algorithms on most of the tasks. The *Att-RNN* model achieved the best accuracy on almost all tasks (except for the TPB-related task, for which it ranked second) and the best micro-average F-score on TPB-attitude classification. The *BERT* model achieved the highest accuracy on the *TPB-related* task, as well as the highest F-1 score on *HBM-barriers*, *HBM-benefits* and *TPB-related* tasks.

BERT, a recent breakthrough in NLP, has advanced state-of-the-art performance in multiple general domain NLP tasks.[18] A few studies in the Twitter domain also show the superiority of BERT over other machine-learning and deep-learning algorithms.[20,21] The present study also shows that the default BERT can achieve performance comparable to the best algorithm (i.e., *Att-RNN*). Recent studies indicate that the transfer learning of BERT in the biomedicine domain can advance existing state-of-the-art performance.[22] It can be expected that the transfer learning of BERT to the Twitter domain can further advance performance on Twitter-related tasks.

© 2020 Du J et al. *JAMA Network Open.*

eMethods Table 1. Comparison on the impact of word embedding algorithms measured by sensitivity, specificity, and accuracy at levels of relevance to the theory (HBM- and TPB-related) and theoretical constructs

	**Sensitivity**					**Specificity**					**Accuracy**				
	GloVe General	FT HPV	GloVe HPV	W2V HPV	Random	GloVe General	FT HPV	GloVe HPV	W2V HPV	Random	GloVe General	FT HPV	GloVe HPV	W2V HPV	Random
HBM-related	0.8061	**0.8072**	0.7991	0.7969	0.7975	0.7985	0.7954	0.7892	**0.8079**	0.7583	**0.8027**	0.8018	0.7946	0.8019	0.7796
HBM Susceptibility	0.7263	0.6889	0.7071	**0.7606**	0.5980	0.9380	**0.9396**	0.9333	0.9165	0.9295	**0.9058**	0.9015	0.8989	0.8928	0.8791
HBM Severity	0.7597	0.7620	0.7938	0.7767	**0.7845**	0.9337	**0.9419**	0.9272	0.9323	0.9130	0.8992	**0.9063**	0.9008	0.9015	0.8876
HBM Benefits	0.7122	**0.7305**	0.6860	0.7280	0.6713	0.9121	**0.9197**	0.9148	0.9133	0.8816	0.8618	**0.8721**	0.8572	0.8667	0.8287
HBM Barriers	0.8867	0.8890	0.8909	**0.8913**	0.8744	0.9041	**0.9219**	0.8983	0.9181	0.8746	0.8959	**0.9063**	0.8948	0.9054	0.8745
TPB-related	**0.9514**	0.9487	0.9471	0.9506	0.9328	0.8690	0.8710	**0.8769**	0.8583	0.8514	0.9237	0.9226	0.9235	0.9196	0.9054
TPB Attitude	N/A										0.7509	**0.7538**	0.7398	0.7534	0.7259

Note: averaged score from 10 experiments

© 2020 Du J et al. *JAMA Network Open.*

eMethods Table 2. Comparison on the impact of word embedding algorithms measured by precision, recall, and F score at levels of relevance to the theory (HBM- and TPB-related) and theoretical constructs

	**Precision**					**Recall**					**F score**				
	GloVe General	FT HPV	GloVe HPV	W2V HPV	Random	GloVe General	FT HPV	GloVe HPV	W2V HPV	Random	GloVe General	FT HPV	GloVe HPV	W2V HPV	Random
HBM-related	0.8274	0.8254	0.8190	**0.8321**	0.7986	0.8061	**0.8072**	0.7991	0.7969	0.7975	**0.8162**	0.8156	0.8087	0.8140	0.7973
HBM Susceptibility	**0.6846**	0.6784	0.6654	0.6252	0.6483	0.7263	0.6889	0.7071	**0.7606**	0.5980	**0.7021**	0.6805	0.6814	0.6837	0.5747
HBM Severity	0.7437	**0.7681**	0.7333	0.7422	0.6936	0.7597	0.7620	**0.7938**	0.7767	0.7845	0.7484	**0.7626**	0.7593	0.7571	0.7345
HBM Benefits	0.7345	**0.7564**	0.7320	0.7395	0.6571	0.7122	**0.7305**	0.6860	0.7280	0.6713	0.7217	**0.7407**	0.7056	0.7323	0.6618
HBM Barriers	0.8941	**0.9123**	0.8882	0.9077	0.8633	0.8867	0.8890	0.8909	**0.8913**	0.8744	0.8898	**0.8999**	0.8890	0.8992	0.8685
TPB-related	0.9350	0.9357	**0.9383**	0.9300	0.9255	**0.9514**	0.9487	0.9471	0.9506	0.9328	**0.9430**	0.9421	0.9427	0.9401	0.9291
TPB Attitude (micro-averaged)	N/A										0.7483	**0.7515**	0.7383	0.7510	0.7240

Note: averaged score from 10 experiments

© 2020 Du J et al. *JAMA Network Open.*

eMethods Table 3. Comparison on the impact of word embedding algorithms measured by precision, recall, and F-score on TPB attitude classification

	Positive			Negative			Neutral			
	Precision	Recall	F-score	Precision	Recall	F-score	Precision	Recall	F-score	Micro-average F-score
GloVe General	**0.7587**	0.7101	0.7326	0.7856	0.8242	**0.8028**	0.7134	0.7077	**0.7093**	0.7483
FT HPV	0.7425	**0.7500**	**0.7447**	0.7987	0.8235	0.8103	**0.7172**	0.6843	0.6996	**0.7515**
GloVe HPV	0.7457	0.7078	0.7257	**0.8016**	0.7871	0.7925	0.6822	**0.7154**	0.6966	0.7383
W2V HPV	0.7460	0.7345	0.7396	0.7977	**0.8264**	0.8108	0.7151	0.6928	0.7025	0.7510
Random	0.7308	0.7028	0.7157	0.7651	0.7892	0.7759	0.6831	0.6794	0.6805	0.7240

Note: averaged score from 10 experiments

© 2020 Du J et al. *JAMA Network Open.*

eMethods Table 4. Comparison of deep learning and machine learning algorithms measured by sensitivity, specificity, and accuracy

	**Sensitivity**					**Specificity**					**Accuracy**				
	Deep learning			Machine learning		Deep learning			Machine learning		Deep learning			Machine learning	
	Att-RNN	BERT	Att-ELMo	TFIDF	Mean-emb	Att-RNN	BERT	Att-ELMo	TFIDF	Mean-emb	Att-RNN	BERT	Att-ELMo	TFIDF	Mean-emb
HBM-related	0.8072	0.8193	**0.8230**	0.7962	0.8176	**0.7954**	0.7678	0.7707	0.7684	0.5378	**0.8018**	0.7958	0.7992	0.7835	0.6900
HBM Susceptibility	0.6889	0.8364	0.8152	**0.8646**	0.7525	**0.9396**	0.8331	0.8651	0.8752	0.7727	**0.9015**	0.8336	0.8575	0.8736	0.7696
HBM Severity	0.7620	0.8085	0.7651	**0.8612**	0.7264	**0.9419**	0.9065	0.9247	0.8967	0.7252	**0.9063**	0.8871	0.8931	0.8897	0.7255
HBM Benefits	0.7305	0.8006	0.6689	**0.8152**	0.6848	0.9197	0.8869	**0.9375**	0.8145	0.7559	**0.8721**	0.8652	0.8699	0.8147	0.7380
HBM Barriers	0.8890	**0.9003**	0.8922	0.8874	0.7327	**0.9219**	0.9099	0.8831	0.9058	0.7764	**0.9063**	0.9054	0.8874	0.8971	0.7557
TPB-related	0.9487	0.9489	0.9294	**0.9526**	0.9521	0.8710	**0.8851**	0.8591	0.8035	0.4092	0.9226	**0.9274**	0.9058	0.9025	0.7696
TPB Attitude	N/A										**0.7538**	0.7423	0.7420	0.7261	0.6178

Note: averaged score from 10 experiments

© 2020 Du J et al. *JAMA Network Open.*

eMethods Table 5. Comparison of deep learning and machine learning algorithms measured by precision, recall, and F score

	**Precision**					**Recall**					**F score**				
	Deep learning			Machine learning		Deep learning			Machine learning		Deep learning			Machine learning	
	Att-RNN	BERT	Att-ELMo	TFIDF	Mean-emb	Att-RNN	BERT	Att-ELMo	TFIDF	Mean-emb	Att-RNN	BERT	Att-ELMo	TFIDF	Mean-emb
HBM-related	**0.8254**	0.8084	0.8108	0.8039	0.6784	0.8072	0.8193	**0.8230**	0.7962	0.8176	0.8156	0.8136	**0.8166**	0.8000	0.7414
HBM Susceptibility	**0.6784**	0.4782	0.5229	0.5555	0.3727	0.6889	0.8364	0.8152	**0.8646**	0.7525	**0.6805**	0.6062	0.6359	0.6758	0.4981
HBM Severity	**0.7681**	0.6837	0.7221	0.6738	0.3951	0.7620	0.8085	0.7651	**0.8612**	0.7264	**0.7626**	0.7394	0.7393	0.7554	0.5115
HBM Benefits	0.7564	0.7070	**0.7876**	0.5968	0.4858	0.7305	0.8006	0.6689	**0.8152**	0.6848	0.7407	**0.7487**	0.7212	0.6888	0.5680
HBM Barriers	**0.9123**	0.9004	0.8734	0.8947	0.7473	0.8890	**0.9003**	0.8922	0.8874	0.7327	0.8999	**0.9002**	0.8825	0.8909	0.7396
TPB-related	0.9357	**0.9424**	0.9288	0.9055	0.7610	0.9487	0.9489	0.9294	**0.9526**	0.9521	0.9421	**0.9455**	0.9291	0.9284	0.8459
TPB Attitude (micro-averaged)	N/A										**0.7515**	0.7409	0.7399	0.7263	0.6138

Note: averaged score from 10 experiments

© 2020 Du J et al. *JAMA Network Open.*

eMethods Table 6. Comparison of deep learning and machine learning algorithms measured by precision, recall, and F-score on TPB attitude classification (average score from 10 experiments)

		Positive			Negative			Neutral			
		Precision	Recall	F-score	Precision	Recall	F-score	Precision	Recall	F-score	Micro-average F-score
Deep learning	Att-RNN	0.7425	**0.7500**	**0.7447**	0.7987	**0.8235**	**0.8103**	**0.7172**	0.6843	0.6996	**0.7515**
	BERT	0.7470	0.7303	0.7372	0.7880	0.8048	0.7958	0.6944	0.6872	0.6898	0.7409
	Att-ELMo	**0.7487**	0.7171	0.7320	0.7750	0.7937	0.7830	0.7053	**0.7070**	**0.7048**	0.7399
Machine learning	TFIDF	0.7315	0.7132	0.7218	**0.8087**	0.7615	0.7838	0.6489	0.7004	0.6732	0.7263
	Mean-emb	0.6144	0.5745	0.5934	0.6475	0.7093	0.6768	0.5858	0.5581	0.5712	0.6138

© 2020 Du J et al. *JAMA Network Open.*

REFERENCES

1. Shah H. Twitter Sentiment Analysis. *Int J Adv Res Comput Sci Softw Eng*. 2018;7(12):15. doi:10.23956/ijarcsse.v7i12.493

2. Zhang L, Wang S, Liu B. Deep learning for sentiment analysis: A survey. *Wiley Interdiscip Rev Data Min Knowl Discov*. 2018;8(4):e1253. doi:10.1002/widm.1253

3. Zhang X, Zhao J, Lecun Y. Character-level convolutional networks for text classification. In: *Advances in Neural Information Processing Systems*. Vol 2015-Janua. ; 2015:649-657.

4. Liu G, Guo J. Bidirectional LSTM with attention mechanism and convolutional layer for text classification. *Neurocomputing*. 2019.

5. Yao L, Mao C, Luo Y. Graph convolutional networks for text classification. In: *Proceedings of the AAAI Conference on Artificial Intelligence*. Vol 33. ; 2019:7370-7377.

6. Word embedding - Wikipedia. https://en.wikipedia.org/wiki/Word_embedding. Accessed July 2, 2020.

7. Word embeddings | TensorFlow Core. https://www.tensorflow.org/tutorials/text/word_embeddings. Accessed June 22, 2020.

8. Zhou P, Shi W, Tian J, et al. Attention-based bidirectional long short-term memory networks for relation classification. In: *54th Annual Meeting of the Association for Computational Linguistics, ACL 2016 - Short Papers*. Vol 2. ; 2016:207-212.

9. Du J, Cunningham RM, Xiang Y, et al. Leveraging deep learning to understand health beliefs about the Human Papillomavirus Vaccine from social media. *npj Digit Med*. 2019;2(1):27.

10. Mikolov T, Sutskever I, Chen K, Corrado G, Dean J. Distributed representations ofwords and phrases and their compositionality. In: *Advances in Neural Information Processing*

© 2020 Du J et al. *JAMA Network Open.*

Systems. ; 2013:3111-3119.

11. Pennington J, Socher R, Manning CD. GloVe: Global vectors for word representation. EMNLP 2014 - 2014 Conference on Empirical Methods in Natural Language Processing, Proceedings of the Conference.

12. Bojanowski P, Grave E, Joulin A, Mikolov T. Enriching Word Vectors with Subword Information. *Trans Assoc Comput Linguist*. 2017;5:135-146.

13. Google Inc. Google Code Archive. Code.Google.com. https://code.google.com/archive/. Published 2016.

14. Roberts K. Assessing the corpus size vs. similarity trade-off for word embeddings in clinical NLP. *Proc Clin Nat Lang Process Work*. 2016:54-63.

15. Geurts P, Ernst D, Wehenkel L. Extremely randomized trees. *Mach Learn*. 2006;63(1):3-42.

16. Ramos J. Using TF-IDF to Determine Word Relevance in Document Queries. In: *Proceedings of the First Instructional Conference on Machine Learning*. Vol 242. ; 2003:133-142.

17. Peters M, Neumann M, Iyyer M, et al. Deep Contextualized Word Representations. *arXiv Prepr arXiv180205365*. 2018:2227-2237.

18. Devlin J, Chang M-W, Lee K, Toutanova K. BERT: Pre-training of Deep Bidirectional Transformers for Language Understanding. *arXiv Prepr arXiv181004805*. 2018.

19. Vaswani A, Shazeer N, Parmar N, et al. Attention is all you need. In: *Advances in Neural Information Processing Systems*. Vol 2017-Decem. ; 2017:5999-6009.

20. Nikolov A, Radivchev V. Nikolov-Radivchev at SemEval-2019 Task 6: Offensive Tweet Classification with BERT and Ensembles. In: *Proceedings of the 13th International*

© 2020 Du J et al. *JAMA Network Open.*

Workshop on Semantic Evaluation. ; 2019:691-695.

21. Ma G. *Tweets Classification with BERT in the Field of Disaster Management*. https://github.com/huggingface/pytorch-pretrained-BERT. Accessed June 18, 2019.

22. Peng Y, Yan S, Lu Z. Transfer Learning in Biomedical Natural Language Processing: An Evaluation of BERT and ELMo on Ten Benchmarking Datasets. *arXiv Prepr arXiv190605474*. 2019:58-65.

© 2020 Du J et al. *JAMA Network Open.*
